# Consequences of exclusion of precipitation on microorganisms and microbial consumers in montane tropical rainforests

**DOI:** 10.1007/s00442-012-2360-6

**Published:** 2012-05-22

**Authors:** Valentyna Krashevska, Dorothee Sandmann, Mark Maraun, Stefan Scheu

**Affiliations:** J.F. Blumenbach Institute of Zoology and Anthropology, Georg August University Göttingen, Berliner Straße 28, 37073 Göttingen, Germany

**Keywords:** Rain exclusion, Testate amoebae, Ergosterol, Microbial biomass, Food web

## Abstract

The structure and functioning of decomposer systems heavily relies on soil moisture. However, this has been primarily studied in temperate ecosystems; little is known about how soil moisture affects the microfaunal food web in tropical regions. This lack of knowledge is surprising, since the microfaunal food web controls major ecosystem processes. To evaluate the role of precipitation in the structure of soil food web components (i.e., microorganisms and testate amoebae), we excluded water input by rain in montane rainforests at different altitudes in Ecuador. Rain exclusion strongly reduced microbial biomass and respiration by about 50 %, and fungal biomass by 23 %. In testate amoebae, rain exclusion decreased the density of live cells by 91 % and caused a shift in species composition at each of the altitudes studied, with ergosterol concentrations, microbial biomass, and water content explaining 25 % of the variation in species data. The results document that reduced precipitation negatively affects soil microorganisms, but that the response of testate amoebae markedly exceeds that of bacteria and fungi. This suggests that, in addition to food, low precipitation directly affects the community structure of testate amoebae, with the effect being more pronounced at lower altitudes. Overall, the results show that microorganisms and testate amoebae rapidly respond to a reduction in precipitation, with testate amoebae—representatives of higher trophic levels—being more sensitive. The results imply that precipitation and soil moisture in tropical rainforests are the main factors regulating decomposition and nutrient turnover.

**Electronic supplementary material:**

The online version of this article (doi:10.1007/s00442-012-2360-6) contains supplementary material, which is available to authorized users.

## Introduction

Species diversity is disproportionaly distributed across the globe, with the highest concentrations found in tropical regions (Dirzo and Raven 2003). The diversity, abundance, and activity of organisms essentially depends on a few fundamental environmental factors such as temperature and moisture (Magurran and May 1999; Gaston and Blackburn 2000). More important than absolute values, however, are variations in these factors. The tolerance of taxa toward varying environmental conditions essentially determines the structure and functioning of communities. In particular, climatic factors such as changes in precipitation drive ecosystem processes by modifying water availability and nutrient cycling. Primary production, but also the decomposition of organic matter and the associated decomposer community responsible for litter breakdown, critically rely on the availability of water. In fact, precipitation has been shown to drive litter mass loss in grasslands, temperate forests, as well as in tropical forests, predominantly by modifying the structure and activity of microbial and animal communities (Swift et al. 1979; Austin and Vitousek 2000; Epstein et al. 2002; Lensing and Wise 2007). In particular, microorganisms and microfauna grazers such as protists and nematodes rely on water films in the soil, and soil humidity therefore plays a predominant role in processes associated with the activity of these organisms, such as litter decomposition and nutrient cycling (Stark and Firestone 1995; Bamforth 2007). Importantly, microorganisms and microfauna are sensitive to changes in soil water concentrations and respond quickly to changes in soil moisture.

Changes in precipitation resulting from regional factors such as deforestation or from global warming and associated changes during El Niño events may strongly affect the intensity of rainfall in tropical forests, and the regional climate may become drier, thereby changing the structure and functioning of these highly diverse ecosystems (Davidson et al. 2004; Beck et al. 2008). Such changes likely also occur in tropical montane forests of the Andes of South America (Pohle 2008). Generally, montane forests of the tropical zone of the Andes are very wet; in forests in southern Ecuador, precipitation increases strongly with altitude—at 3,000 m it exceeds 4,000 mm per year (Moser et al. 2007). High precipitation may positively affect the diversity of plants, but on the other hand it is associated with water-logged soils and low oxygen supply (Liptzin et al. 2010), which negatively affects decomposers, resulting in slow decomposition of litter materials and thick organic layers (Wilcke et al. 2002). Decomposer communities in wet, oxygen-poor ecosystems with high organic matter contents are colonized predominantly by microorganisms and microfauna, including protists, especially testate amoebae (Schönborn 1973; Schaefer 1991; Krashevska et al. 2007; Smith et al. 2008). By consuming a wide range of food (i.e., bacteria, fungi, algae, other protists, and some small metazoans), testate amoebae are assumed to play an important role in carbon and nutrient cycling and other ecological processes (Gilbert et al. 2000; Krashevska et al. 2010; Wilkinson and Mitchell 2010).

The composition of testate amoeba communities has been assumed to be driven mainly by environmental factors, particularly abiotic factors, most importantly soil moisture, pH, light, temperature, nutrient availability, and oxygen concentration (Schönborn 1962; Corbet 1973; Lousier 1974; Meisterfeld 1977; Charman 1997; Mitchell et al. 2004; Mieczan 2009; Krashevska et al. 2010). In addition, biotic factors such as microbial food resources have been proposed to structure testate amoeba community composition (Krashevska et al. 2008). Unfortunately, inferences on the structuring forces of testate amoeba communities are based almost exclusively on correlative evidence; experimental manipulations of abiotic and biotic factors are lacking almost entirely. Therefore, the factors structuring testate amoeba communities are largely hypothetical, and this applies in particular to those of tropical ecosystems.

To better understand the role of precipitation and soil moisture as a structuring force for testate amoeba communities of tropical rainforests, we experimentally excluded precipitation. To relate changes in the testate amoeba community to changes in their prey, we also analyzed the response of soil microorganisms. In testate amoebae, we investigated both the response of live cells and their decay-resistent shells, which serve as bioindicators of environmental conditions at recent but also geological timescales (Charman 1997; Smith et al. 2008; Mieczan 2009). The experiment was set up along an altitudinal transect from 1,000 to 2,000 to 3,000 m a.s.l., i.e., along a gradient of strongly increasing precipitation. We expected (1) the effects of exclusion of precipitation to increase with altitude (i.e., to be most pronounced in the wettest systems), and (2) the response of testate amoebae to be linked closely to that of microorganisms, as their major prey. To trace the mechanisms responsible for changes in microorganisms and testate amoebae, we analyzed abiotic factors which likely change with reduced precipitation.

## Materials and methods

### Study sites

The study sites are located in southern Ecuador at the northern border of the Podocarpus National Park on the eastern slopes of the Andes. Three study sites along an altitudinal transect were selected at 1,000, 2,000, and 3,000 m a.s.l. The tropical montane rainforests of Ecuador are semihumid, with 8–10 humid months per year. Annual rainfall is high and increases from 2,200 to 3,500 to 4,500 mm year^−1^ at 1,000, 2,000, and 3,000 m, respectively. The mean annual air temperature decreases with altitude from 19.4 to 15.7 to 9.4 °C at 1,000, 2,000, and 3,000 m, respectively. The coldest month on average is August, while the warmest is November (Moser et al. 2007; Beck et al. 2008). More details of the study sites are given in Beck et al. (2008); details on testate amoebae and their interrelationships with abiotic and biotic factors are given in Krashevska et al. (2007, 2008).

### Experimental design and sampling

Precipitation was excluded from replicated plots at each altitudinal site, with the exclusion maintained from July 2007 to October 2008. Experimental plots were established by installing translucent polyester roofs of an area of 1.5 × 1.5 m. To prevent mass flow of water in the upper soil layers into the plots, plastic barriers were dug into the soil at the hillside of the plot. An equivalent number of control plots of the same size were established by erecting roofs covered with mesh (5 × 5 mm), allowing free water entry but excluding litter input, as in the rain exclusion plots. Control plots were located in the immediate vicinity of the rain exclusion plots. Rainfall exclusion and control plots were arranged in four blocks (randomized complete block design). Four replicates were set up at each altitude (1,000, 2,000, and 3,000 m), resulting in eight plots per altitude.

After 15 months (29 September 2008), samples from the litter/fermentation layer (L/F) were taken from each plot to a depth of 5 cm using a corer (∅ 5 cm), resulting in 24 samples in total. Three corers were taken per plot and pooled. The material consisted of decomposing leaves, seeds, flowers, twigs, fine roots, and some large woody material. Samples were transferred to the laboratory at the nearby research station. From these samples, subsamples of ca. 100 g were taken, placed in plastic bags, stored in a refrigerator (5 °C) for a maximum of two days, and transported in cooled boxes to Germany, where they were processed immediately for analysis of testate amoebae, microbial biomass, and ergosterol concentration.

### Environmental factors

Using the samples, a set of environmental factors that likely affect testate amoebae were investigated. Litter/fermentation layer material was milled, dried at 65 °C for 72 h, and analyzed for total C and N concentrations using an elemental analyzer (Carlo Erba, Milan, Italy). Further, pH(CaCl_2_) was measured using a digital pH-meter. Water content was determined gravimetrically from five samples taken at regular intervals during the 15 months of the experiment.

To inspect potential differences in light intensity and temperature in rain exclusion and control plots, the photosynthetically active radiation and temperature were measured at the soil surface using a LI-250A light meter (LI-COR Inc., Lincoln, NE, USA) and a digital thermometer, respectively. Mean temperature and light intensity were calculated from five measurements taken at regular intervals during the 15 months of the experiment, including rainy and sunny days.

### Microorganisms

Microbial respiration and biomass were determined by measuring O_2_ consumption using an automated respirometer system (Scheu 1992). Microbial basal respiration of moist field samples was measured at 22 °C as the mean O_2_ consumption during hours 10–20 after attachment to the respirometer. Microbial biomass *C* (*C*_mic_; μg g^−1^ dry weight) was assessed by measuring the maximum initial respiratory response (MIRR; μl O_2_ g^−1^ h^−1^) after glucose addition at 22 °C, and calculated as 38× MIRR (SIR method; Anderson and Domsch 1978; Beck et al. 1997). Glucose (80 mg g^−1^ litter dry weight) was added as an aqueous solution, adjusting the water content to 80–90 % of the water-holding capacity of the L/F material (Joergensen and Scheu 1999). The mean of the three lowest measurements during the first 10 h after glucose addition was taken as the MIRR.

Ergosterol concentration was determined according to the method described in Djajakirana et al. (1996). Ergosterol concentrations were measured by reversed-phase high-performance liquid chromatography (System Gold 125, Beckman Coulter, Fullerton, CA, USA) using the following setup: main column 10 cm, pre-column 0.5 cm (Spherisorb ODS II, 5 μm diameter), mobile phase 100 % methanol, flow rate 1.0 ml h^−1^, and detection at 282 nm (System Gold 166 UV detector, Beckman Coulter).

### Testate amoebae

Testate amoebae were extracted by washing samples over a filter of 500 μm mesh and then back-sieving the filtrate over a 20 μm mesh. Microscopic slides were prepared and tests were identified and counted at 200× and 400× magnification with an upright Leitz Ortholux II and a Nikon inverted microscope (DIAPHOT-TMD). Testate amoebae were divided into live cells, cysts, and empty shells after staining with aniline blue. To stain the testate amoebae on microscopic slides, one drop of aniline blue solution (2 %) was added (see Schönborn 1968). Determination of species was based on morphological characters (morphospecies). More details of the identification and taxonomic references are presented in Krashevska et al. (2007). Full names of species are listed in alphabetical order in online resource 1 of the Electronic supplementary material (ESM).

### Statistical analysis

Data on testate amoebae (species number, density of live cells, cysts, and empty shells), microbial parameters (microbial basal respiration, microbial biomass, ergosterol concentration), and environmental factors (C-to-N ratio, pH, water content, light intensity, temperature) were analyzed by two-factor randomized complete block analysis of variance (ANOVA) with the fixed factors rain exclusion (with and without) and altitude (1,000, 2,000 and 3,000 m). Data on water content were arcsine square-root transformed prior to statistical analyses. Tukey′s HSD test (*α* < 0.05) was used to identify significant differences between means.

The fixed factors which affected the species composition of testate amoebae were identified using MANOVA. To identify which of the species were responsible for the significant MANOVA effects, protected ANOVAs were carried out, and Pearson correlation coefficients were used to identify whether the effects were positive or negative. Data on the density of testate amoebae were log(*x* + 1) transformed. In addition, Pearson correlation coefficients were used to investigate correlations between environmental factors and microbial parameters (Scheiner and Gurevich 2001). Statistical analyses were performed using SAS 9.13 (SAS Institute Inc., Cary, NC, USA) and STATISTICA 7.0 for Windows (StatSoft, Tulsa, OK, USA).

Data on live cells of testate amoebae were analyzed by discriminant function analysis (DFA) to identify treatment effects. DFA was carried out using STATISTICA 7.0. Squared Mahalanobis distances between group centroids (control and rain exclusion treatments) and the reliability of sample classification were determined. Two significant canonical roots were derived and graphically presented in two-dimensional space to show significant differences between control and rain exclusion treatments at 1,000, 2,000, and 3,000 m.

Relationships between live cells of testate amoeba communities and environmental factors were analyzed using redundancy analysis (RDA) as implemented in CANOCO (Ter Braak 1988–1992). Redundancy analysis was chosen since the length of the gradient was between three and four (Lepš and Šmilauer 2003). Redundancy analysis allows dependent variables (species of testate amoeba) to be related to a set of independent variables (environmental conditions) by direct ordination. Environmental variables included only environmental factors which were significantly affected by rain exclusion and/or the rain exclusion × altitude interaction. Since the L/F material of the three altitudinal sites contained different amounts of mineral soil, which heavily influences the soil water content based on percentage dry weight, we expressed the water content as the percentage of the amount of carbon in the L/F layer. Only species occurring in at least two replicates were included in the RDA. Monte Carlo tests (999 permutations) were performed to evaluate the significance of individual axes (Ter Braak 1996). Treatment levels (control and rain exclusion as well as 1,000, 2,000, and 3,000 m) were included as passive variables.

## Results

### Environmental factors

Water content in the L/F layer was significantly reduced in rain exclusion treatments (*F*_1,23_ = 81.4, *P* < 0.0001; Table 1). However, changes in water content between control and rain exclusion plots differed between altitudes (*F*_2,23_ = 10.19, *P* = 0.005 for the rain exclusion × altitude interaction; Table 1); differences were more pronounced at 1,000 than at 2,000 and 3,000 m. The C-to-N ratio of the L/F material significantly increased with increasing altitude (*F*_2,23_ = 13.99, *P* = 0.0017); it was slightly higher in rain exclusion plots at 1,000 and 2,000 and markedly higher at 3,000 m (Table 1). Light intensity in control plots generally exceeded that in rain exclusion plots; however, differences varied between altitudinal sites and were most pronounced at 3,000 m (*F*_2,23_ = 5.19, *P* = 0.03 for the rain exclusion × altitude interaction; Table 1). The pH in the L/F layer was significantly increased by rain exclusion at 1,000 m, whereas it was slightly decreased at 2,000 and 3,000 m (*F*_2,23_ = 7.42, *P* = 0.01 for the rain exclusion × altitude interaction; Table 1). Generally, the pH in the L/F layer significantly decreased with altitude in the order 1,000 < 2,000 ≤ 3,000 m (*F*_2,23_ = 18.61, *P* = 0.0006; Table 1). Forest floor temperature was not significantly affected by rain exclusion, but significantly decreased with altitude (*F*_1,23_ = 40.6, *P* < 0.001; Table 1).

**Table 1:** Effect of rain exclusion on environmental factors, microorganisms, and density of testate amoebae in control (contr) and rain exclusion treatments (rexcl) at three altitudes (1000, 2000, and 3000 m)

	1,000contr		1,000rexcl		2,000contr		2,000rexcl		3,000contr		3,000rexcl	
	Mean	SD	Mean	SD	Mean	SD	Mean	SD	Mean	SD	Mean	SD
*Environmental factors*												
Litter water content (% of litter dry weight)	175	82 ab	20	10 a	495	76 c	208	83 b	451	94 c	241	98 b
Litter C-to-N ratio	20	2 a	21	3 a	31	3 ab	33	3 ab	32	5 ab	42	11 b
Light intensity (μMol s^−1^m^−2^)	9	3 bc	2	0 c	19	15 b	6	4 bc	187	165 a	14	3 b
Litter pH (CaCl_2_)	4.0	0.3 b	4.8	0.5 a	3.9	0.2 b	3.7	0.3 b	3.6	0.5 b	3.4	0.1 b
Forest floor temperature (°C)	19.6	0.1 a	19.9	0.3 a	20.6	1.8 a	19.8	1.0 a	10.4	0.5 b	10.6	0.7 b
*Microorganisms*												
Microbial basal respiration (μl O_2_ g^−1^ litter dry weight h^−1^)	88	29 cd	14	3 a	82	6 bcd	49	11 ab	106	1 d	55	24 bc
Microbial biomass (mg *C*_mic_ g^−1^ litter dry weight)	11	3 c	4	2 a	9	4 ab	5	2 a	14	2 c	7	1 ab
Ergosterol concentration (μg g^−1^ litter dry weight)	40	6 a	32	10 a	51	8 a	33	16 a	114	3 b	93	4 c
*Density of testate amoebae*												
Live cells (ind. g^−1^ litter dry weight)	1,194	645 a	10	20 b	1,263	609 a	87	95 b	1,364	357 a	243	156 b
Cysts (ind. g^−1^ litter dry weight)	55	102 a	14	27 a	0	0 a	193	22 a	12	14 a	392	193 b
Empty shells (ind. g^−1^ litter dry weight)	5,678	3,587 a	1,268	986 a	4,012	1,689 a	4,422	3,407 a	1,905	727 a	3,555	1,224 a

Means with SD (*n* = 4); treatments with different letters vary significantly (Tukey’s HSD test,* α* < 0.05)

Pearson correlation coefficients indicated that the C-to-N ratio was negatively correlated with pH (*r* = −0.8, *P* = 0.0001) and positively correlated with the water content (*r* = 0.7, *P* = 0.0001). Further, pH was negatively correlated with water content (*r* = −0.6, *P* = 0.0001). In addition, temperature was negatively correlated with C-to-N ratio (*r* = −0.6, *P* = 0.001) and water content (*r* = −0.5, *P* = 0.05), and positively correlated with pH (*r* = 0.8, *P* < 0.0001).

### Microorganisms

Generally, microbial basal respiration (BR) in rain exclusion plots was reduced to 43 % of that in control plots (*F*_1,23_ = 55.3, *P* < 0.0001; Table 1). However, differences in BR in rain exclusion plots significantly decreased with increasing altitude (*F*_2,23_ = 8.2, *P* = 0.009 for the rain exclusion × altitude interaction; Table 1). Similar to BR, microbial biomass *C* (*C*_mic_) in rain exclusion plots was on average only 50 % of that in control plots (*F*_1,23_ = 39.0, *P* < 0.0001; Table 1). Further, *C*_mic_ significantly varied with altitude and increased in the order 2,000 < 1,000 < 3,000 m (*F*_2,23_ = 6.0, *P* = 0.02). Pearson correlation coefficients indicated that *C*_mic_ increased with increasing water content (*r* = 0.5, *P* = 0.007) and increasing light intensity (*r* = 0.4, *P* = 0.04), but decreased with increasing temperature (*r* = −0.4, *P* = 0.04). Exclusion of rain significantly decreased the ergosterol concentration to 77 % of that in control plots (*F*_1,23_ = 23.0, *P* = 0.001; Table 1). Further, ergosterol concentrations significantly increased with increasing altitude in the order 1,000 ≤ 2,000 < 3,000 m (*F*_2,23_ = 163.9, *P* < 0.0001; Table 1). Pearson correlation coefficients indicated that ergosterol concentrations increased with increasing litter C-to-N ratio (*r* = 0.6, *P* = 0.001), but decreased with increasing pH (*r* = −0.8, *P* = 0.0001) and increasing temperature (*r* = −0.9, *P* < 0.0001).

### Testate amoebae

A total of 112 taxa of testate amoebae were identified (see online resource 1 of the ESM). In general, species diversity was high at 2,000 (81 taxa) and 1,000 m (79 taxa), and lower at 3,000 m (66 taxa). Rain exclusion did not affect the total number of species of testate amoebae, but mean species numbers of live cells increased significantly with altitude in the order 1,000 < 2,000 < 3,000 m from 10 to 17 to 26 species, respectively (*F*_2,23_ = 30.6, *P* < 0.001). In contrast to species numbers, rain exclusion significantly decreased the density of live cells to 8.8 % of that in control plots, and explained 77 % of the variance (*F*_1,23_ = 40.3, *P* < 0.001; Table 1), whereas altitude explained only 13 %, and the interaction of both explained 10 %. Further, the density of cysts of testate amoebae significantly increased in rain exclusion plots, but only at 2,000 and 3,000 m (*F*_2,23_ = 12.5, *P* = 0.0025 for the rain exclusion × altitude interaction; Table 1). Rain exclusion explained 39 % of the variance in cyst density, altitude explained 23 %, and the interaction of both explained 37 %. At 1,000 m, the density of empty shells in control treatments exceeded that in rain exclusion plots, whereas the opposite was true at 2,000 and in particular at 3,000 m (*F*_1,23_ = 4.10, *P* = 0.03 for the rain exclusion × altitude interaction; Table 1). Only 4 % of the variance in empty shell density was explained by rain exclusion, 71 % was explained by altitude, and 25 % was explained by the interaction of both.

MANOVA suggested that the community structure of live cells of testate amoebae significantly responded to rain exclusion, but the response varied with altitude (Wilks’* λ* = 0.188, *F*_4,18_ = 5.89, *P* = 0.003 for the rain exclusion × altitude interaction). Protected ANOVAs and Pearson correlation coefficients suggest that, in total, 43 species (of 66 live taxa) of testate amoebae significantly responded to rain exclusion, altitude, or the interaction between these factors (see online resource 2 of the ESM). In contrast, none of these factors nor their interaction affected the number of cysts of testate amoeba species.

**Fig. 1:**
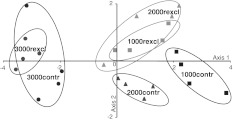
Discriminant function analysis of live cells of testate amoeba along the altitudinal transect (*axis 1*) and between control and rain exclusion treatments (*axis 2*). Control and rain exclusion treatments at 1,000 (*1,000contr*,* 1,000rexcl*), 2,000 (*2,000contr*,* 2,000rexcl*), and 3,000 m (*3,000contr*,* 3,000rexcl*);* ellipses* represent confidence ranges at* α* = 0.05

For live cells, discriminant function analysis separated testate amoeba communities of the different altitudinal sites (axis 1), but also the communities of control treatments and rain exclusion treatments (axis 2; Wilks’* λ* = 0.087, *F*_10,34_ = 8.08, *P* < 0.001; Fig. 1). Generally, communities of testate amoebae at 3,000 m differed markedly from those at 2,000 and 1,000 m. Rain exclusion caused a shift in the composition of testate amoebae at 1,000 and 2,000, but not at 3,000 m; however, communities in rain exclusion plots at 3,000 m became less variable than in control plots (Fig. 1; Table 2).

**Table 2:** Squared Mahalanobis distances between group centroids and reliability of discrimination based on the density of live cells of testate amoebae in control (contr) and rain exclusion treatments (rexcl) at three altitudes (1,000, 2,000, and 3,000 m)

	1,000contr	1,000rexcl	2,000contr	2,000rexcl	3,000contr
1,000rexcl	4.4*	–			
2,000contr	3.5*	2.5	–		
2,000rexcl	5.2*	0.3	4.4*	–	
3,000contr	24.5**	10.8**	10.2**	12.9**	–
3,000rexcl	33.9**	16.1**	17.1**	18.1**	0.9

* *P* < 0.05, ** *P* < 0.005

**Fig. 2:**
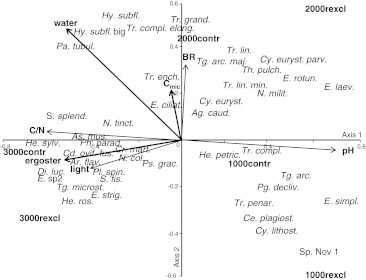
RDA ordination diagram of live cells of testate amoebae with environmental variables. Significant factors represented by* bold arrows* (*ergoster* fungal biomass, *C*_mic_ microbial biomass,* water* litter water content). Variables with an explanatory power of less than 5 % are represented by* non-bold arrows* (*BR* microbial basal respiration,* pH* litter pH,* light* light intensity,* C/N* litter C-to-N ratio). Control and rain exclusion treatments at 1,000 (*1,000contr*,* 1,000rexcl*), 2,000 (*2,000contr*,* 2,000rexcl*) and 3,000 m (*3,000contr*,* 3,000rexcl*) were included as passive variables. *Axes 1* and *2* explained 15 and 8 % of the variation in species data, respectively. Full species names are given in online resource 1 of the ESM

In the forward selection procedure of RDA, three of the seven quantitative explanatory variables (see “Materials and methods”) were significantly related to the community of live testate amoebae (*P* < 0.05; Fig. 2). Together, these variables explained 41 % of the variation in species data, with the trace being significant (*F* = 1.60, *P* = 0.002). Ergosterol concentration accounted for most of the variation in species data (11 % of total; *F* = 2.72, *P* = 0.001). The second environmental variable with significant explanatory power was *C*_mic_ (accounting for an additional 7 % of the variation; *F* = 1.89, *P* = 0.009), and the third was the water content (accounting for another 7 % of the variation; *F* = 1.61, *P* = 0.02). The remaining 16 % of the variation were explained by variables with an explanatory power of less than 5 %, i.e., microbial basal respiration (4.5 %), pH (4 %), light intensity (4 %), and litter C-to-N ratio (3.5 %).

The position of testate amoebae in the ordination diagram reflects the higher abundance and diversity in the control as compared to the rain exclusion treatments (Fig. 2, online resource 1 in the ESM). Rain exclusion alone and in combination with other factors affected 25 live taxa (protected ANOVAs and Pearson correlations, see online resource 2 in the ESM). *Cyclopyxis eurystoma* *parvula*, *Heleopera petricola*, *Nebela militaris*, *Plagiopyxis declivis*, *Trachelocorythion pulchellum*, and *Trinema grandis* were associated with control treatments, indicating that they suffered from rain exclusion, whereas rain exclusion negatively affected *Euglypha rotunda*, *Euglypha* *laevis*, and *Tr*. *complanatum*, with the effect for the first two being most pronounced at 2,000 and for the latter at 1,000 m (Fig. 2; see online resources 1 and 2 of the ESM).

Axis 1 of the RDA was negatively correlated with ergosterol concentration, litter C-to-N ratio, and light intensity, and was positively correlated with pH, whereas axis 2 was positively correlated with water content and *C*_mic_. The ordination indicates that the increased abundance and diversity in the control treatments was more pronounced at 1,000 and 2,000 than at 3,000 m. At 3,000 m, the species in control and rain exclusion plots differed little, but their abundance was lower in rain exclusion plots. Some species that were present in rain exclusion plots at 3,000 m occurred only in control treatments at 1,000 and 2,000 m, including *Euglypha strigosa*, *Phryganella paradoxa*, and *Sphenoderia splendida* (Fig. 2; online resource 1 in the ESM). Generally, ergosterol concentration, light intensity, and litter C-to-N ratio pointed in a similar direction and were greatest at 3,000 m. A large number of species clustered at this site, including *He*. *sylvatica*, *Assulina muscorum*, *Difflugia lucida*, *E*. sp2, *Trigonopyxis microstoma*, *He*. *rosea,* *Cryptodifflugia oviformis* *fusca*, *Archerella flavum*, *E*. *strigosa*, and *Placocista spinosa*. At 2,000 m, the differences between control and rain exclusion treatments were more pronounced, with most species associated with the control treatment and high water content, including *Hyalosphenia subflava* (big), *Padaungiella tubulata*, *Hy*. *subflava*, and *Tr*. *complanatum* *elongata*. Also, at 1,000 m, most species were associated with the control sites, including *Tg. arcula*, *Pg. declivis*, *Tr*. *penardi*, *Centropyxis* *plagiostoma*, and *Cy*. *lithostoma*. Only Sp. Nov 1 was most closely associated with the rain exclusion at 1,000 m (Fig. 2; see online resource 1 in the ESM). Furthermore, the preferential occurrence of taxa such as *He*. *sylvatica*, *N*. *collaris*, *Cd*. *oviformis* *fusca*, *As*. *muscorum*, *He*. *rosea*, *N*. *tincta*, *Ph*. *paradoxa*, *S*. *splendida*, and *Certesella martiali* at 3,000 m correlated with high ergosterol concentrations. In contrast, the occurrence of *Argynnia caudata*, *E*. *ciliata*, *Cy*. *eurystoma*, *Tr*. *enchelys*, and *Tg*. *arcula major* showed the closest correlation with *C*_mic_. The occurrences of *Ar*. *flavum* and *Pl*. *spinosa* were closely correlated with light intensity (Fig. 2; see online resource 2 in the ESM).

## Discussion

The structure of testate amoeba communities and decomposition processes are assumed to be driven mainly by abiotic factors, but this has been little studied using experimental manipulations of climatic factors such as precipitation. Lousier (1974) showed that adding water to aspen woodland soil in Canada increased the number of testate amoebae. Investigating wet tropical forests, Schuur (2001) found that increased rainfall negatively affected litter decomposition, which contrasts with results obtained using soils of the temperate zone (Borken et al. 2006). The role of precipitation in the decomposer community in tropical montane rainforests had not previously been investigated. Therefore, we experimentally excluded precipitation in montane rainforests at different altitudes to evaluate the role of precipitation in the structure of soil food web components that rely heavily on soil moisture (i.e., microorganisms and testate amoebae).

### Microorganisms

As hypothesized, the exclusion of precipitation negatively affected soil microorganisms. Basal respiration and microbial biomass generally declined in rain exclusion plots; however, effects of control and rain exclusion plots differed between altitudes. At 1,000 m in rain exclusion treatments, basal respiration decreased to 15 % of that in control plots, whereas at 2,000 and 3,000 m, basal respiration decreased to only 41 and 48 % of that in the control plots, respectively. Microbial biomass in rain exclusion plots responded in parallel, with the reduction being most pronounced at 1,000 m (to 39 % of that in control plots) and less at 2,000 (45 %) and 3,000 m (48 %). This suggests that microorganisms at low altitudes are more sensitive to reduced precipitation than those at higher altitudes. However, changes in water content between rain exclusion and control plots were most pronounced at 1,000 m, presumably due to the generally lower precipitation at 1,000 as compared to 2,000 and 3,000 m (Beck et al. 2008).

Similar to microbial biomass, ergosterol concentrations generally decreased with rain exclusion but increased with altitude, presumably due to decreasing pH and increasing C-to-N ratio (Djajakirana et al. 1996; Frostegård and Bååth 1996; Bååth and Anderson 2003). In contrast to basal respiration and microbial biomass, excluding precipitation reduced ergosterol concentrations the most at 3,000 m. This is likely related to the shift from a bacteria-dominated system at 1,000 m to a fungi-dominated system at higher altitudes, in particular at 3,000 m (Krashevska et al. 2008). Overall, the results of the present study document that precipitation plays an important role for microorganisms, with bacteria being most strongly affected at low altitudes and fungi at higher altitudes (i.e., bacteria and fungi were affected most at the sites at which they were most dominant).

### Testate amoebae

We expected the response of testate amoebae to be closely linked to that of microorganisms. The results only partly supported this hypothesis, since rain exclusion more strongly affected testate amoebae than microorganisms, explaining 77 % of the variance of live cell density. At 1,000 m, in rain exclusion treatments, the density of live testate amoebae decreased to less than 1 % of that in control plots, at 2,000 m to 6 %, and at 3,000 m to 17 %. Therefore, the community structure of testate amoebae is unlikely to be driven only by changes in microbial community structure and activity; rather, rain exclusion likely also directly affected testate amoebae.

Species number of live cells increased significantly with increasing altitude, independent of rain exclusion. In contrast to the number of species, the density of live cells significantly decreased with the exclusion of precipitation, irrespective of altitude. This underlines that the animals depend on water for movement and feeding (Sleigh 1989). However, exclusion of precipitation also excludes the input of nutrients such as N, Ca, Mg, P, and K in throughfall (Wilcke et al. 2008), and this may have contributed to the reduced density of testate amoebae in rain exclusion plots. Exclusion of precipitation was further associated with increased numbers of cysts, reflecting unfavorable conditions for testate amoebae. Generally, the results suggest that virtually all testate amoeba species at the study sites respond negatively to the exclusion of precipitation, with precipitation playing a major role for at least 37 % of the taxa.

Exclusion of precipitation and associated changes in abiotic and biotic factors also affected the composition of the community of testate amoebae, but this was restricted to 1,000 and 2,000 m, whereas the community compositions of the control and rain exclusion plots were similar at 3,000 m. To identify the factors responsible for the changes in the community structure of testate amoebae, correlations between abiotic and biotic factors and the density of individual taxa of testate amoebae were investigated. Of 66 live taxa, the densities of 43 were significantly correlated with at least one of the measured abiotic or biotic factors. In most of these species, one or several factors associated with rain exclusion contributed to the reduction in density.

As indicated by correlations between *C*_mic_ and the density of testate amoebae taxa, the availability of bacteria and fungi may limit the density of *Ag*. *caudata*, *E*. *ciliata*, *Cy*. *eurystoma*, *Tr*. *enchelys*, and *Tg*. *arcula major*. Correlations with ergosterol concentrations suggest that a number of species rely on fungi as food, including *He*. *sylvatica*, *N*. *collaris*, *Cd*. *oviformis fusca*, *As*. *muscorum*, *He*. *rosea*, *N*. *tincta*, *Ph*. *paradoxa*, *S*. *splendida*, and *Cr*. *martiali*, which is consistent with earlier suggestions regarding the feeding preferences of testate amoebae (Gilbert et al. 2000; Krashevska et al. 2010; Wilkinson and Mitchell 2010). However, correlations of amoebae density with ergosterol concentrations need not necessarily indicate trophic interrelationships; rather, they may be driven by factors associated with increased fungal biomass, such as fungal exudates and bacteria associated with fungal mycelia (Vohnik et al. 2011).

Water content was also significantly correlated with testate amoeba species, and the variation explained by water content and *C*_mic_ suggests that both of these variables were of similar importance. Taxa such as *P*. *tubulata*, *N*. *tincta,* *As*. *muscorum*, *E*. *strigosa*, and *S*. *splendida* were also significantly affected by water content, which is likely related to the aperture morphology with less protected from drying terminally located aperture, i.e., acrostomic pseudostoms. The high similarity of testate amoebae community composition and the dominance of taxa with acrostomic pseudostoms at 3,000 m are likely related to the fact that the high water content differed little between the control and rain exclusion treatments. This contrasted with the pattern at 2,000 and in particular at 1,000 m, where soil moisture was lower and morphotypes that were more protected from drying were more abundant, such as those with cryptostomic, cyclostomic, and plagiostomic pseudostoms.

Some species were positively correlated with light intensity, likely reflecting the light limitations of mixotroph species containing symbiotic algae, such as *Ar*. *flavum* and *Pl*. *spinosa* (Schönborn 1965; Meisterfeld 1977).

Generally, at 3,000 m, testate amoebae taxa were most affected by soil water content, ergosterol content, litter C-to-N ratio, light intensity, and low pH; at 2,000 m they were most affected by water content and microbial biomass, and at 1,000 m, they were most affected by pH, litter C-to-N ratio, and water content.

In conclusion, the results of the present study document that excluding precipitation negatively affects soil microorganisms in tropical montane rainforests. However, unexpectedly, the response of testate amoebae was stronger than that of bacteria and fungi, suggesting that reduced precipitation not only affects testate amoebae via reduced food supply, but also directly, for example by restricting their movement and altering the dominance of morphotypes. Further, the results show that microorganisms and the microbial food web in general, including testate amoebae, respond rapidly to reduced precipitation, suggesting that short-term fluctuations in soil moisture alter decomposition and nutrient turnover in tropical rainforests. Notably, testate amoebae representing higher trophic levels responded more sensitively than microorganisms as basal consumers of the microbial food web. Reduced grazing on soil microorganisms by testate amoebae under drier climatic conditions is likely to aggravate nutrient deficiency and nutrient limitation, and thereby reduce ecosystem productivity. Knowledge of the sensitivity of microorganisms and microbial grazers to changes in precipitation is needed to understand the responses of ecosystems to anthropogenic and natural climate changes.

## Electronic supplementary material

Below is the link to the electronic supplementary material.

**Online resource 1.** Species list and density of testate amoebae in experimental treatments (*Contr* control,* rexcl* rain exclusion) and altitudes (1000, 2000, and 3000 m) in the studied tropical montane rainforest of Ecuador.

**Online resource 2.** Variations in the abundance of live cells of testate amoeba taxa with rain exclusion and altitude in tropical montane rainforests (fitted individually; protected ANOVAs), and correlations with biotic and abiotic factors associated with rain exclusion and altitude (Pearson correlation). For authorities of species, see online resource 1.

Supplementary material 1 (DOC 463 kb)

Supplementary material 2 (DOC 133 kb)
