# Supplementary material for: Consequences of exclusion of precipitation on microorganisms and microbial consumers in montane tropical rainforests
Source: Oecologia. 2012 May 22;170(4):1067–76. doi: 10.1007/s00442-012-2360-6 (PMC3496542; doi:10.1007/s00442-012-2360-6)
Supplement: Supplementary file 1 — Supplementary material 1 (DOC 463 kb) [file 442_2012_2360_MOESM1_ESM.doc]

**Consequences of exclusion of precipitation on microorganisms and microbial** **consumers in montane tropical rainforests**

Valentyna Krashevska, Dorothee Sandmann, Mark Maraun and Stefan Scheu

J.F. Blumenbach Institute of Zoology and Anthropology, Georg August University Göttingen, Berliner Straße 28, 37073 Göttingen, Germany

Author for correspondence

Valentyna Krashevska

Mail [vkrashe@gwdg.de](mailto:vkrashe@gwdg.de)

Tel: +49 (0) 551 395557

Fax: +49 (0) 551 395448

**Online resource 1.** Species list and density of testate amoebae in experimental treatments (*Contr* control, *rexcl* rain exclusion) and altitudes (1000, 2000 and 3000 m) in the studied tropical montane rainforests of Ecuador.

| Taxa | 1000 m contr | | | | | | 1000 m rexcl | | | | | | 2000 m contr | | | | | | 2000 m rexcl | | | | | | 3000 m contr | | | | | | 3000 m rexcl | | | | | |
| --- | --- | --- | --- | --- | --- | --- | --- | --- | --- | --- | --- | --- | --- | --- | --- | --- | --- | --- | --- | --- | --- | --- | --- | --- | --- | --- | --- | --- | --- | --- | --- | --- | --- | --- | --- | --- |
|  | live cells | | cysts | | empty shells | | live cells | | cysts | | empty shells | | live cells | | cysts | | empty shells | | live cells | | cysts | | empty shells | | live cells | | cysts | | empty shells | | live cells | | cysts | | empty shells | |
|  | Mean | SD | Mean | SD | Mean | SD | Mean | SD | Mean | SD | Mean | SD | Mean | SD | Mean | SD | Mean | SD | Mean | SD | Mean | SD | Mean | SD | Mean | SD | Mean | SD | Mean | SD | Mean | SD | Mean | SD | Mean | SD |
| *Apodera vas* Certes, 1889 | 0 | 0 | 0 | 0 | 0 | 0 | 0 | 0 | 0 | 0 | 0 | 0 | 8 | 8 | 0 | 0 | 8 | 8 | 0 | 0 | 0 | 0 | 14 | 14 | 7 | 7 | 0 | 0 | 13 | 7 | 0 | 0 | 0 | 0 | 0 | 0 |
| *Arcella arenaria* Greeff, 1866 | 27 | 16 | 0 | 0 | 10 | 10 | 0 | 0 | 0 | 0 | 0 | 0 | 0 | 0 | 0 | 0 | 4 | 4 | 0 | 0 | 0 | 0 | 0 | 0 | 7 | 7 | 0 | 0 | 0 | 0 | 0 | 0 | 0 | 0 | 0 | 0 |
| *Argynnia caudata* Leidy, 1879 | 41 | 29 | 0 | 0 | 43 | 16 | 0 | 0 | 0 | 0 | 3 | 3 | 31 | 31 | 0 | 0 | 35 | 30 | 0 | 0 | 7 | 7 | 136 | 81 | 172 | 127 | 0 | 0 | 19 | 12 | 0 | 0 | 0 | 0 | 0 | 0 |
| *Argynnia dentistoma* Penard, 1890 | 0 | 0 | 0 | 0 | 41 | 18 | 0 | 0 | 0 | 0 | 0 | 0 | 0 | 0 | 0 | 0 | 25 | 4 | 0 | 0 | 0 | 0 | 46 | 37 | 0 | 0 | 0 | 0 | 13 | 7 | 0 | 0 | 0 | 0 | 0 | 0 |
| *Argynnia spicata* Wailes, 1913 | 0 | 0 | 0 | 0 | 0 | 0 | 0 | 0 | 0 | 0 | 0 | 0 | 0 | 0 | 0 | 0 | 8 | 8 | 0 | 0 | 0 | 0 | 0 | 0 | 0 | 0 | 0 | 0 | 0 | 0 | 0 | 0 | 0 | 0 | 0 | 0 |
| *Argynnia vitraea* Penard, 1899 | 0 | 0 | 0 | 0 | 0 | 0 | 0 | 0 | 0 | 0 | 0 | 0 | 0 | 0 | 0 | 0 | 0 | 0 | 0 | 0 | 0 | 0 | 13 | 13 | 0 | 0 | 0 | 0 | 0 | 0 | 0 | 0 | 0 | 0 | 0 | 0 |
| *Archerella flavum* Archer, 1877 | 0 | 0 | 0 | 0 | 0 | 0 | 0 | 0 | 0 | 0 | 0 | 0 | 20 | 15 | 0 | 0 | 0 | 0 | 0 | 0 | 0 | 0 | 0 | 0 | 12 | 7 | 0 | 0 | 0 | 0 | 46 | 28 | 0 | 0 | 0 | 0 |
| *Assulina muscorum* Greef, 1888 | 4 | 4 | 10 | 10 | 44 | 30 | 0 | 0 | 0 | 0 | 3 | 3 | 9 | 5 | 0 | 0 | 62 | 13 | 0 | 0 | 0 | 0 | 114 | 67 | 58 | 24 | 0 | 0 | 31 | 12 | 14 | 14 | 0 | 0 | 109 | 29 |
| *Assulina scandinavica* Penard, 1890 | 0 | 0 | 0 | 0 | 0 | 0 | 0 | 0 | 0 | 0 | 0 | 0 | 0 | 0 | 0 | 0 | 25 | 4 | 0 | 0 | 0 | 0 | 15 | 9 | 0 | 0 | 0 | 0 | 13 | 7 | 0 | 0 | 0 | 0 | 0 | 0 |
| *Bullinularia indica* (Penard, 1911) Deflandre, 1953 | 0 | 0 | 0 | 0 | 7 | 7 | 0 | 0 | 0 | 0 | 0 | 0 | 0 | 0 | 0 | 0 | 0 | 0 | 0 | 0 | 0 | 0 | 0 | 0 | 0 | 0 | 0 | 0 | 0 | 0 | 0 | 0 | 0 | 0 | 0 | 0 |
| *Centropyxis aculeata* (Ehrenberg, 1838) Stein, 1857 | 0 | 0 | 0 | 0 | 7 | 7 | 0 | 0 | 0 | 0 | 0 | 0 | 0 | 0 | 0 | 0 | 0 | 0 | 0 | 0 | 0 | 0 | 0 | 0 | 0 | 0 | 0 | 0 | 0 | 0 | 0 | 0 | 0 | 0 | 0 | 0 |
| *Centropyxis aerophila* Deflandre, 1929 | 0 | 0 | 0 | 0 | 54 | 5 | 0 | 0 | 0 | 0 | 5 | 3 | 0 | 0 | 0 | 0 | 13 | 8 | 0 | 0 | 0 | 0 | 13 | 13 | 0 | 0 | 0 | 0 | 7 | 7 | 0 | 0 | 0 | 0 | 0 | 0 |
| *Centropyxis cassis* (Wallich, 1864) Deflandre, 1929 | 0 | 0 | 0 | 0 | 30 | 24 | 0 | 0 | 0 | 0 | 13 | 13 | 9 | 9 | 0 | 0 | 5 | 5 | 0 | 0 | 0 | 0 | 20 | 13 | 0 | 0 | 0 | 0 | 0 | 0 | 0 | 0 | 0 | 0 | 0 | 0 |
| *Centropyxis constricta* (Ehrenberg, 1841) Deflandre, 1929 | 47 | 35 | 0 | 0 | 100 | 50 | 0 | 0 | 0 | 0 | 55 | 29 | 36 | 26 | 0 | 0 | 93 | 66 | 0 | 0 | 0 | 0 | 47 | 37 | 0 | 0 | 0 | 0 | 7 | 7 | 0 | 0 | 0 | 0 | 0 | 0 |
| *Centropyxis ecornis* (Ehrenberg, 1841) Leidy, 1879 | 0 | 0 | 0 | 0 | 18 | 18 | 0 | 0 | 0 | 0 | 0 | 0 | 0 | 0 | 0 | 0 | 0 | 0 | 0 | 0 | 0 | 0 | 0 | 0 | 0 | 0 | 0 | 0 | 0 | 0 | 0 | 0 | 0 | 0 | 0 | 0 |
| *Centropyxis elongata* (Penard, 1890) Thomas, 1959 | 0 | 0 | 0 | 0 | 185 | 51 | 0 | 0 | 0 | 0 | 27 | 13 | 0 | 0 | 0 | 0 | 5 | 5 | 0 | 0 | 0 | 0 | 0 | 0 | 0 | 0 | 0 | 0 | 0 | 0 | 0 | 0 | 0 | 0 | 0 | 0 |
| *Centropyxis orbicularis* Deflandre, 1929 | 0 | 0 | 0 | 0 | 0 | 0 | 0 | 0 | 0 | 0 | 0 | 0 | 0 | 0 | 0 | 0 | 0 | 0 | 0 | 0 | 0 | 0 | 0 | 0 | 7 | 7 | 0 | 0 | 12 | 7 | 0 | 0 | 0 | 0 | 0 | 0 |
| *Centropyxis plagiostoma* Bonnet, Thomas, 1955 | 5 | 11 | 0 | 0 | 48 | 35 | 0 | 0 | 0 | 0 | 9 | 6 | 0 | 0 | 0 | 0 | 0 | 0 | 0 | 0 | 0 | 0 | 0 | 0 | 0 | 0 | 0 | 0 | 0 | 0 | 0 | 0 | 0 | 0 | 0 | 0 |
| *Centropyxis sylvatica* (Deflandre, 1929) Bonnet, Thomas, 1955 | 0 | 0 | 0 | 0 | 11 | 11 | 0 | 0 | 0 | 0 | 0 | 0 | 0 | 0 | 0 | 0 | 0 | 0 | 0 | 0 | 0 | 0 | 0 | 0 | 0 | 0 | 0 | 0 | 0 | 0 | 0 | 0 | 0 | 0 | 0 | 0 |
| *Certesella martiali* Certes, 1889 | 0 | 0 | 0 | 0 | 13 | 13 | 0 | 0 | 0 | 0 | 0 | 0 | 5 | 9 | 0 | 0 | 0 | 0 | 0 | 0 | 13 | 13 | 0 | 0 | 12 | 7 | 0 | 0 | 37 | 22 | 0 | 0 | 0 | 0 | 47 | 30 |
| *Cornuapyxis lunaristoma* Couteaux, Chardez, 1981 | 0 | 0 | 0 | 0 | 0 | 0 | 0 | 0 | 0 | 0 | 0 | 0 | 0 | 0 | 0 | 0 | 28 | 22 | 0 | 0 | 0 | 0 | 13 | 13 | 0 | 0 | 0 | 0 | 0 | 0 | 0 | 0 | 0 | 0 | 0 | 0 |
| *Corythion asperulum* Schonborn, 1988 | 0 | 0 | 0 | 0 | 0 | 0 | 0 | 0 | 0 | 0 | 0 | 0 | 0 | 0 | 0 | 0 | 0 | 0 | 0 | 0 | 0 | 0 | 0 | 0 | 0 | 0 | 0 | 0 | 0 | 0 | 0 | 0 | 0 | 0 | 31 | 31 |
| *Corythion dubium* Taranek, 1871 | 0 | 0 | 0 | 0 | 0 | 0 | 0 | 0 | 0 | 0 | 0 | 0 | 48 | 97 | 0 | 0 | 65 | 43 | 0 | 0 | 0 | 0 | 34 | 13 | 61 | 54 | 0 | 0 | 44 | 12 | 15 | 15 | 31 | 18 | 129 | 56 |
| *Cryptodifflugia compressa* Penard, 1902 | 0 | 0 | 0 | 0 | 0 | 0 | 0 | 0 | 0 | 0 | 0 | 0 | 0 | 0 | 0 | 0 | 9 | 9 | 0 | 0 | 0 | 0 | 0 | 0 | 0 | 0 | 0 | 0 | 0 | 0 | 0 | 0 | 0 | 0 | 0 | 0 |
| *Cryptodifflugia oviformis* *fusca* Penard, 1890 | 0 | 0 | 0 | 0 | 0 | 0 | 0 | 0 | 0 | 0 | 0 | 0 | 8 | 16 | 0 | 0 | 21 | 15 | 0 | 0 | 0 | 0 | 8 | 8 | 30 | 12 | 0 | 0 | 37 | 7 | 0 | 0 | 29 | 29 | 30 | 17 |
| *Cyclopyxis ambigua* Bonnet, Thomas, 1960 | 0 | 0 | 0 | 0 | 22 | 13 | 0 | 0 | 0 | 0 | 26 | 19 | 0 | 0 | 0 | 0 | 36 | 16 | 0 | 0 | 0 | 0 | 0 | 0 | 0 | 0 | 0 | 0 | 0 | 0 | 0 | 0 | 0 | 0 | 0 | 0 |
| *Cyclopyxis arcelloides* (Penard, 1902) Deflandre, 1929 | 0 | 0 | 0 | 0 | 0 | 0 | 0 | 0 | 0 | 0 | 0 | 0 | 0 | 0 | 0 | 0 | 0 | 0 | 0 | 0 | 0 | 0 | 0 | 0 | 5 | 5 | 0 | 0 | 5 | 5 | 14 | 14 | 0 | 0 | 14 | 14 |
| *Cyclopyxis eurystoma* Deflandre, 1929 | 16 | 20 | 0 | 0 | 188 | 75 | 0 | 0 | 0 | 0 | 46 | 25 | 9 | 10 | 0 | 0 | 143 | 22 | 0 | 0 | 0 | 0 | 179 | 68 | 5 | 5 | 0 | 0 | 35 | 14 | 0 | 0 | 0 | 0 | 158 | 77 |
| *Cyclopyxis eurystoma* *parvula* Bonnet, Thomas, 1960 | 153 | 125 | 0 | 0 | 970 | 390 | 0 | 0 | 0 | 0 | 187 | 40 | 102 | 57 | 0 | 0 | 503 | 114 | 0 | 0 | 8 | 8 | 642 | 299 | 17 | 11 | 0 | 0 | 37 | 16 | 0 | 0 | 0 | 0 | 175 | 74 |
| *Cyclopyxis kahli* Deflandre, 1929 | 0 | 0 | 0 | 0 | 36 | 6 | 0 | 0 | 0 | 0 | 7 | 7 | 29 | 46 | 0 | 0 | 96 | 46 | 13 | 13 | 0 | 0 | 142 | 57 | 0 | 0 | 0 | 0 | 6 | 6 | 0 | 0 | 0 | 0 | 15 | 15 |
| *Cyclopyxis lithostoma* Bonnet, 1974 | 16 | 24 | 0 | 0 | 112 | 34 | 3 | 3 | 0 | 0 | 56 | 20 | 0 | 0 | 0 | 0 | 0 | 0 | 0 | 0 | 0 | 0 | 7 | 7 | 0 | 0 | 0 | 0 | 0 | 0 | 0 | 0 | 0 | 0 | 0 | 0 |
| *Cyclopyxis puteus* Thomas, 1960 | 0 | 0 | 0 | 0 | 0 | 0 | 0 | 0 | 0 | 0 | 3 | 3 | 0 | 0 | 0 | 0 | 0 | 0 | 0 | 0 | 0 | 0 | 21 | 13 | 0 | 0 | 0 | 0 | 0 | 0 | 0 | 0 | 0 | 0 | 0 | 0 |
| *Difflugia lucida* Penard, 1890 | 0 | 0 | 0 | 0 | 13 | 13 | 0 | 0 | 0 | 0 | 7 | 7 | 0 | 0 | 0 | 0 | 29 | 20 | 0 | 0 | 0 | 0 | 8 | 8 | 11 | 11 | 0 | 0 | 36 | 14 | 0 | 0 | 0 | 0 | 32 | 19 |
| *Difflugia oblonga* Ehrenberg, 1838 | 0 | 0 | 0 | 0 | 0 | 0 | 0 | 0 | 0 | 0 | 0 | 0 | 5 | 9 | 0 | 0 | 9 | 9 | 0 | 0 | 0 | 0 | 0 | 0 | 0 | 0 | 0 | 0 | 0 | 0 | 0 | 0 | 0 | 0 | 0 | 0 |
| *Difflugia penardi* Hopkinson, 1909 | 0 | 0 | 0 | 0 | 0 | 0 | 0 | 0 | 0 | 0 | 0 | 0 | 0 | 0 | 0 | 0 | 0 | 0 | 0 | 0 | 0 | 0 | 0 | 0 | 0 | 0 | 0 | 0 | 6 | 6 | 0 | 0 | 0 | 0 | 0 | 0 |
| *Euglypha anodonta* Bonnet, 1960 | 0 | 0 | 0 | 0 | 21 | 21 | 0 | 0 | 0 | 0 | 0 | 0 | 0 | 0 | 0 | 0 | 0 | 0 | 0 | 0 | 0 | 0 | 0 | 0 | 0 | 0 | 0 | 0 | 0 | 0 | 0 | 0 | 0 | 0 | 0 | 0 |
| *Euglypha ciliata* (Ehrenber, 1848) Leidy, 1878 | 0 | 0 | 0 | 0 | 0 | 0 | 0 | 0 | 0 | 0 | 0 | 0 | 0 | 0 | 0 | 0 | 4 | 4 | 0 | 0 | 0 | 0 | 0 | 0 | 6 | 6 | 0 | 0 | 25 | 11 | 0 | 0 | 0 | 0 | 0 | 0 |
| *Euglypha compressa* Carter, 1864 | 13 | 25 | 0 | 0 | 21 | 12 | 0 | 0 | 0 | 0 | 12 | 7 | 0 | 0 | 0 | 0 | 37 | 10 | 0 | 0 | 0 | 0 | 7 | 7 | 0 | 0 | 0 | 0 | 17 | 10 | 0 | 0 | 0 | 0 | 0 | 0 |
| *Euglypha compressa* *glabra* Wailes, 1915 | 0 | 0 | 0 | 0 | 0 | 0 | 0 | 0 | 0 | 0 | 5 | 5 | 0 | 0 | 0 | 0 | 0 | 0 | 0 | 0 | 0 | 0 | 0 | 0 | 0 | 0 | 0 | 0 | 0 | 0 | 0 | 0 | 0 | 0 | 0 | 0 |
| *Euglypha capsiosa* Couteaux, 1978 | 0 | 0 | 0 | 0 | 0 | 0 | 0 | 0 | 0 | 0 | 0 | 0 | 4 | 9 | 0 | 0 | 42 | 15 | 0 | 0 | 0 | 0 | 74 | 48 | 7 | 7 | 0 | 0 | 0 | 0 | 15 | 15 | 0 | 0 | 0 | 0 |
| *Euglypha cristata* Leidy, 1874 | 0 | 0 | 0 | 0 | 11 | 11 | 0 | 0 | 0 | 0 | 0 | 0 | 20 | 31 | 0 | 0 | 70 | 23 | 0 | 0 | 0 | 0 | 7 | 7 | 0 | 0 | 0 | 0 | 13 | 13 | 0 | 0 | 0 | 0 | 0 | 0 |
| *Euglypha cristata* *major* Wailes, 1912 | 10 | 21 | 0 | 0 | 21 | 21 | 0 | 0 | 0 | 0 | 0 | 0 | 0 | 0 | 0 | 0 | 13 | 13 | 0 | 0 | 0 | 0 | 26 | 15 | 0 | 0 | 0 | 0 | 13 | 7 | 0 | 0 | 0 | 0 | 0 | 0 |
| *Euglypha cristata* *decora* Jung, 1942 | 0 | 0 | 0 | 0 | 0 | 0 | 0 | 0 | 0 | 0 | 0 | 0 | 0 | 0 | 0 | 0 | 8 | 8 | 0 | 0 | 0 | 0 | 0 | 0 | 0 | 0 | 0 | 0 | 0 | 0 | 0 | 0 | 0 | 0 | 0 | 0 |
| *Euglypha denticulata* Brown, 1912 | 0 | 0 | 0 | 0 | 0 | 0 | 0 | 0 | 0 | 0 | 3 | 3 | 0 | 0 | 0 | 0 | 0 | 0 | 0 | 0 | 0 | 0 | 0 | 0 | 0 | 0 | 0 | 0 | 0 | 0 | 0 | 0 | 0 | 0 | 0 | 0 |
| *Euglypha filifera* Penard, 1890 | 0 | 0 | 0 | 0 | 0 | 0 | 0 | 0 | 0 | 0 | 0 | 0 | 0 | 0 | 0 | 0 | 0 | 0 | 0 | 0 | 0 | 0 | 0 | 0 | 0 | 0 | 0 | 0 | 12 | 7 | 0 | 0 | 0 | 0 | 14 | 14 |
| *Euglypha hyalina* Couteaux, 1978 | 0 | 0 | 0 | 0 | 10 | 10 | 0 | 0 | 0 | 0 | 0 | 0 | 0 | 0 | 0 | 0 | 0 | 0 | 0 | 0 | 0 | 0 | 0 | 0 | 0 | 0 | 0 | 0 | 0 | 0 | 0 | 0 | 0 | 0 | 0 | 0 |
| *Euglypha laevis* (Ehrenberg, 1832) Perty, 1849 | 83 | 57 | 0 | 0 | 244 | 140 | 3 | 3 | 0 | 0 | 8 | 5 | 87 | 45 | 0 | 0 | 141 | 75 | 0 | 0 | 0 | 0 | 60 | 34 | 12 | 12 | 0 | 0 | 0 | 0 | 0 | 0 | 0 | 0 | 14 | 14 |
| *Euglypha polylepis* Bonnet, Thomas, 1960 | 0 | 0 | 0 | 0 | 0 | 0 | 0 | 0 | 0 | 0 | 5 | 5 | 0 | 0 | 0 | 0 | 8 | 8 | 0 | 0 | 0 | 0 | 13 | 13 | 0 | 0 | 0 | 0 | 0 | 0 | 0 | 0 | 0 | 0 | 0 | 0 |
| *Euglypha rotunda* Wailes, Penard, 1911 | 9 | 11 | 0 | 0 | 71 | 47 | 0 | 0 | 0 | 0 | 7 | 7 | 20 | 15 | 0 | 0 | 62 | 23 | 0 | 0 | 0 | 0 | 13 | 13 | 0 | 0 | 0 | 0 | 0 | 0 | 0 | 0 | 0 | 0 | 0 | 0 |
| *Euglypha simplex* Decloitre, 1965 | 158 | 116 | 4 | 4 | 433 | 83 | 0 | 0 | 8 | 8 | 153 | 36 | 8 | 16 | 0 | 0 | 37 | 9 | 13 | 13 | 13 | 13 | 71 | 36 | 0 | 0 | 0 | 0 | 7 | 7 | 0 | 0 | 0 | 0 | 32 | 19 |
| *Euglypha strigosa* (Ehrenberg, 1871) Leidy, 1878 | 23 | 27 | 0 | 0 | 14 | 14 | 0 | 0 | 0 | 0 | 3 | 3 | 64 | 109 | 0 | 0 | 49 | 38 | 0 | 0 | 0 | 0 | 37 | 23 | 102 | 39 | 0 | 0 | 96 | 34 | 32 | 32 | 17 | 17 | 128 | 52 |
| *Euglypha strigosa* *glabra* Wailes, 1898 | 45 | 82 | 10 | 10 | 83 | 42 | 0 | 0 | 0 | 0 | 8 | 5 | 0 | 0 | 0 | 0 | 21 | 13 | 0 | 0 | 0 | 0 | 14 | 14 | 7 | 7 | 0 | 0 | 36 | 12 | 0 | 0 | 0 | 0 | 157 | 95 |
| *Euglypha* sp1 | 0 | 0 | 0 | 0 | 0 | 0 | 0 | 0 | 0 | 0 | 0 | 0 | 0 | 0 | 0 | 0 | 8 | 8 | 0 | 0 | 0 | 0 | 13 | 13 | 0 | 0 | 0 | 0 | 6 | 6 | 0 | 0 | 0 | 0 | 0 | 0 |
| *Euglypha* sp2 | 0 | 0 | 0 | 0 | 0 | 0 | 0 | 0 | 0 | 0 | 0 | 0 | 0 | 0 | 0 | 0 | 0 | 0 | 0 | 0 | 0 | 0 | 0 | 0 | 5 | 5 | 0 | 0 | 0 | 0 | 0 | 0 | 0 | 0 | 0 | 0 |
| *Heleopera petricola* Leidy, 1879 | 37 | 60 | 10 | 10 | 213 | 50 | 0 | 0 | 0 | 0 | 66 | 31 | 32 | 25 | 0 | 0 | 70 | 22 | 0 | 0 | 0 | 0 | 113 | 85 | 26 | 19 | 7 | 7 | 40 | 17 | 0 | 0 | 43 | 43 | 191 | 150 |
| *Heleopera petricola* *amethystea* Penard, 1902 | 0 | 0 | 0 | 0 | 43 | 8 | 0 | 0 | 0 | 0 | 22 | 12 | 0 | 0 | 0 | 0 | 0 | 0 | 0 | 0 | 0 | 0 | 0 | 0 | 0 | 0 | 0 | 0 | 0 | 0 | 0 | 0 | 0 | 0 | 0 | 0 |
| *Heleopera petricola humicola* Bonnet, Thomas, 1955 | 0 | 0 | 0 | 0 | 0 | 0 | 0 | 0 | 0 | 0 | 0 | 0 | 4 | 9 | 0 | 0 | 20 | 15 | 0 | 0 | 0 | 0 | 39 | 25 | 0 | 0 | 0 | 0 | 13 | 13 | 0 | 0 | 0 | 0 | 14 | 14 |
| *Heleopera rosea* Penard, 1890 | 0 | 0 | 0 | 0 | 4 | 4 | 0 | 0 | 0 | 0 | 0 | 0 | 0 | 0 | 0 | 0 | 8 | 8 | 0 | 0 | 0 | 0 | 7 | 7 | 42 | 21 | 0 | 0 | 24 | 10 | 0 | 0 | 64 | 64 | 96 | 96 |
| *Heleopera sphagni* Leidy, 1874 | 0 | 0 | 0 | 0 | 10 | 10 | 0 | 0 | 0 | 0 | 0 | 0 | 0 | 0 | 0 | 0 | 0 | 0 | 0 | 0 | 0 | 0 | 0 | 0 | 0 | 0 | 0 | 0 | 0 | 0 | 0 | 0 | 0 | 0 | 0 | 0 |
| *Heleopera sylvatica* Penard, 1890 | 0 | 0 | 0 | 0 | 56 | 33 | 0 | 0 | 0 | 0 | 43 | 22 | 0 | 0 | 0 | 0 | 24 | 13 | 13 | 13 | 0 | 0 | 26 | 15 | 44 | 20 | 0 | 0 | 50 | 35 | 0 | 0 | 0 | 0 | 51 | 51 |
| *Hyalosphenia elegans* Leidy, 1879 | 0 | 0 | 0 | 0 | 36 | 18 | 0 | 0 | 0 | 0 | 8 | 8 | 0 | 0 | 0 | 0 | 0 | 0 | 0 | 0 | 0 | 0 | 0 | 0 | 0 | 0 | 0 | 0 | 0 | 0 | 0 | 0 | 0 | 0 | 0 | 0 |
| *Hyalosphenia minuta* Cash, 1891 | 0 | 0 | 0 | 0 | 10 | 10 | 0 | 0 | 0 | 0 | 0 | 0 | 0 | 0 | 0 | 0 | 0 | 0 | 0 | 0 | 0 | 0 | 0 | 0 | 0 | 0 | 0 | 0 | 0 | 0 | 0 | 0 | 0 | 0 | 0 | 0 |
| *Hyalosphenia ovalis* Wailes, 1912 | 0 | 0 | 0 | 0 | 0 | 0 | 0 | 0 | 0 | 0 | 0 | 0 | 0 | 0 | 0 | 0 | 8 | 8 | 0 | 0 | 0 | 0 | 7 | 7 | 0 | 0 | 0 | 0 | 0 | 0 | 0 | 0 | 0 | 0 | 29 | 29 |
| *Hyalosphenia subflava* Cash, 1909 | 10 | 21 | 0 | 0 | 49 | 27 | 0 | 0 | 3 | 3 | 3 | 3 | 41 | 26 | 0 | 0 | 227 | 117 | 20 | 13 | 27 | 16 | 305 | 144 | 31 | 11 | 0 | 0 | 97 | 37 | 0 | 0 | 32 | 32 | 159 | 70 |
| *Hyalosphenia subflava* (big; 90-100 µm) | 0 | 0 | 0 | 0 | 0 | 0 | 0 | 0 | 0 | 0 | 0 | 0 | 111 | 126 | 0 | 0 | 186 | 58 | 13 | 13 | 7 | 7 | 597 | 432 | 25 | 11 | 5 | 5 | 18 | 6 | 30 | 17 | 31 | 18 | 213 | 93 |
| *Nebela barbata* Leidy, 1874 | 0 | 0 | 0 | 0 | 0 | 0 | 0 | 0 | 0 | 0 | 0 | 0 | 0 | 0 | 0 | 0 | 0 | 0 | 0 | 0 | 8 | 8 | 23 | 15 | 0 | 0 | 0 | 0 | 0 | 0 | 0 | 0 | 0 | 0 | 0 | 0 |
| *Nebela bohemica* Taranek, 1882 | 0 | 0 | 0 | 0 | 0 | 0 | 0 | 0 | 0 | 0 | 0 | 0 | 0 | 0 | 0 | 0 | 0 | 0 | 0 | 0 | 0 | 0 | 13 | 13 | 0 | 0 | 0 | 0 | 0 | 0 | 0 | 0 | 0 | 0 | 0 | 0 |
| *Nebela collaris* (Ehrenberg, 1848) Leidy, 1879 | 0 | 0 | 0 | 0 | 0 | 0 | 0 | 0 | 0 | 0 | 0 | 0 | 5 | 9 | 0 | 0 | 20 | 8 | 0 | 0 | 0 | 0 | 0 | 0 | 18 | 12 | 0 | 0 | 44 | 12 | 0 | 0 | 0 | 0 | 34 | 34 |
| *Nebela galeata* Penard, 1902 | 0 | 0 | 0 | 0 | 0 | 0 | 0 | 0 | 0 | 0 | 5 | 5 | 0 | 0 | 0 | 0 | 0 | 0 | 0 | 0 | 0 | 0 | 0 | 0 | 0 | 0 | 0 | 0 | 0 | 0 | 0 | 0 | 0 | 0 | 0 | 0 |
| *Nebela gracilis* Penard, 1910 | 21 | 42 | 10 | 10 | 114 | 68 | 0 | 0 | 0 | 0 | 19 | 12 | 0 | 0 | 0 | 0 | 25 | 9 | 0 | 0 | 13 | 13 | 91 | 48 | 0 | 0 | 0 | 0 | 26 | 19 | 0 | 0 | 0 | 0 | 46 | 46 |
| *Nebela militaris* Penard, 1890 | 52 | 105 | 0 | 0 | 112 | 89 | 0 | 0 | 0 | 0 | 5 | 3 | 60 | 61 | 0 | 0 | 43 | 38 | 0 | 0 | 0 | 0 | 73 | 29 | 17 | 10 | 0 | 0 | 43 | 31 | 0 | 0 | 0 | 0 | 47 | 30 |
| *Nebela parvula* Cash, 1909 | 0 | 0 | 0 | 0 | 10 | 10 | 0 | 0 | 0 | 0 | 0 | 0 | 0 | 0 | 0 | 0 | 0 | 0 | 0 | 0 | 0 | 0 | 0 | 0 | 0 | 0 | 0 | 0 | 0 | 0 | 0 | 0 | 0 | 0 | 0 | 0 |
| *Nebela tincta* (Leidy, 1879) Awerintzew, 1906 | 0 | 0 | 0 | 0 | 10 | 10 | 0 | 0 | 0 | 0 | 0 | 0 | 12 | 15 | 0 | 0 | 9 | 5 | 0 | 0 | 0 | 0 | 48 | 23 | 35 | 14 | 0 | 0 | 80 | 22 | 0 | 0 | 32 | 19 | 125 | 4 |
| *Padaungiella lageniformi*s (Penard, 1890) Lara, Todorov, 2011 | 0 | 0 | 0 | 0 | 159 | 91 | 0 | 0 | 0 | 0 | 12 | 6 | 12 | 15 | 0 | 0 | 61 | 23 | 0 | 0 | 0 | 0 | 93 | 44 | 0 | 0 | 0 | 0 | 18 | 12 | 0 | 0 | 0 | 0 | 49 | 31 |
| *Padaungiella tubulata* (Brown, 1911) Lara, Todorov, 2011 | 0 | 0 | 0 | 0 | 13 | 13 | 0 | 0 | 0 | 0 | 0 | 0 | 8 | 16 | 0 | 0 | 59 | 43 | 0 | 0 | 0 | 0 | 42 | 18 | 12 | 7 | 0 | 0 | 25 | 19 | 0 | 0 | 0 | 0 | 77 | 30 |
| *Phryganella paradoxa* Penard, 1902 | 21 | 42 | 0 | 0 | 212 | 125 | 0 | 0 | 0 | 0 | 36 | 17 | 5 | 9 | 0 | 0 | 50 | 28 | 0 | 0 | 0 | 0 | 105 | 64 | 36 | 12 | 0 | 0 | 118 | 68 | 17 | 17 | 0 | 0 | 160 | 44 |
| *Placocista spinosa* (Carter, 1865) Leidy, 1879 | 0 | 0 | 0 | 0 | 0 | 0 | 0 | 0 | 0 | 0 | 0 | 0 | 0 | 0 | 0 | 0 | 0 | 0 | 0 | 0 | 0 | 0 | 0 | 0 | 31 | 23 | 0 | 0 | 23 | 9 | 0 | 0 | 0 | 0 | 79 | 17 |
| *Plagiopyxis callida* Penard, 1910 | 5 | 11 | 0 | 0 | 54 | 36 | 0 | 0 | 0 | 0 | 29 | 26 | 8 | 16 | 0 | 0 | 20 | 15 | 0 | 0 | 0 | 0 | 85 | 36 | 0 | 0 | 0 | 0 | 0 | 0 | 0 | 0 | 0 | 0 | 0 | 0 |
| *Plagiopyxis declivis* Thomas, 1958 | 22 | 21 | 0 | 0 | 63 | 31 | 0 | 0 | 0 | 0 | 33 | 16 | 9 | 18 | 0 | 0 | 41 | 16 | 0 | 0 | 0 | 0 | 36 | 14 | 0 | 0 | 0 | 0 | 0 | 0 | 0 | 0 | 0 | 0 | 0 | 0 |
| *Plagiopyxis labiata* Penard, 1910 | 0 | 0 | 0 | 0 | 5 | 5 | 0 | 0 | 0 | 0 | 3 | 3 | 0 | 0 | 0 | 0 | 0 | 0 | 0 | 0 | 0 | 0 | 13 | 13 | 0 | 0 | 0 | 0 | 0 | 0 | 0 | 0 | 0 | 0 | 0 | 0 |
| *Plagiopyxis minuta* Bonnet, 1959 | 0 | 0 | 0 | 0 | 0 | 0 | 0 | 0 | 0 | 0 | 3 | 3 | 0 | 0 | 0 | 0 | 0 | 0 | 0 | 0 | 0 | 0 | 0 | 0 | 0 | 0 | 0 | 0 | 0 | 0 | 0 | 0 | 0 | 0 | 0 | 0 |
| *Planhoogenraadia acuta* Bonnet, 1977 | 0 | 0 | 0 | 0 | 21 | 21 | 0 | 0 | 0 | 0 | 0 | 0 | 0 | 0 | 0 | 0 | 0 | 0 | 0 | 0 | 0 | 0 | 0 | 0 | 0 | 0 | 0 | 0 | 0 | 0 | 0 | 0 | 0 | 0 | 0 | 0 |
| *Playfairina valkanovi* Golemansky, 1966 | 0 | 0 | 0 | 0 | 4 | 4 | 0 | 0 | 0 | 0 | 0 | 0 | 17 | 13 | 0 | 0 | 46 | 12 | 0 | 0 | 0 | 0 | 0 | 0 | 0 | 0 | 0 | 0 | 12 | 7 | 0 | 0 | 0 | 0 | 0 | 0 |
| *Porosia bigibossa* Penard, 1890 | 0 | 0 | 0 | 0 | 26 | 20 | 0 | 0 | 0 | 0 | 5 | 5 | 12 | 15 | 0 | 0 | 0 | 0 | 0 | 0 | 0 | 0 | 0 | 0 | 0 | 0 | 0 | 0 | 0 | 0 | 0 | 0 | 0 | 0 | 0 | 0 |
| *Pseudodifflugia gracilis* Schlumberger, 1845 | 0 | 0 | 0 | 0 | 0 | 0 | 0 | 0 | 0 | 0 | 0 | 0 | 12 | 15 | 0 | 0 | 89 | 44 | 0 | 0 | 0 | 0 | 14 | 14 | 17 | 10 | 0 | 0 | 53 | 22 | 0 | 0 | 14 | 14 | 47 | 16 |
| *Quadrulella quadrigera* Deflandre, 1936 | 0 | 0 | 0 | 0 | 55 | 17 | 0 | 0 | 0 | 0 | 0 | 0 | 0 | 0 | 0 | 0 | 0 | 0 | 0 | 0 | 0 | 0 | 0 | 0 | 0 | 0 | 0 | 0 | 0 | 0 | 0 | 0 | 0 | 0 | 0 | 0 |
| *Quadrulella symmetrica* (Wallich, 1863) Schulze, 1875 | 16 | 24 | 0 | 0 | 38 | 22 | 0 | 0 | 0 | 0 | 5 | 5 | 0 | 0 | 0 | 0 | 0 | 0 | 0 | 0 | 0 | 0 | 0 | 0 | 0 | 0 | 0 | 0 | 0 | 0 | 0 | 0 | 0 | 0 | 0 | 0 |
| *Schonbornia humicola* Schoenborn, 1964 | 0 | 0 | 0 | 0 | 0 | 0 | 0 | 0 | 0 | 0 | 0 | 0 | 0 | 0 | 0 | 0 | 17 | 17 | 0 | 0 | 0 | 0 | 0 | 0 | 0 | 0 | 0 | 0 | 0 | 0 | 0 | 0 | 0 | 0 | 0 | 0 |
| *Schwabia terricola* Bonnet, Thomas, 1955 | 0 | 0 | 0 | 0 | 0 | 0 | 0 | 0 | 0 | 0 | 0 | 0 | 0 | 0 | 0 | 0 | 8 | 8 | 0 | 0 | 0 | 0 | 0 | 0 | 0 | 0 | 0 | 0 | 0 | 0 | 0 | 0 | 0 | 0 | 0 | 0 |
| *Sphenoderia fissirostris* Penard, 1890 | 0 | 0 | 0 | 0 | 0 | 0 | 0 | 0 | 0 | 0 | 0 | 0 | 8 | 16 | 0 | 0 | 55 | 8 | 0 | 0 | 0 | 0 | 15 | 9 | 12 | 7 | 0 | 0 | 18 | 12 | 0 | 0 | 17 | 17 | 49 | 32 |
| *Sphenoderia minuta* Deflandre, 1931 | 0 | 0 | 0 | 0 | 13 | 13 | 0 | 0 | 0 | 0 | 7 | 7 | 0 | 0 | 0 | 0 | 0 | 0 | 0 | 0 | 0 | 0 | 40 | 40 | 0 | 0 | 0 | 0 | 6 | 6 | 0 | 0 | 0 | 0 | 125 | 72 |
| *Sphenoderia rhombophora* Bonnet, 1966 | 0 | 0 | 0 | 0 | 4 | 4 | 0 | 0 | 0 | 0 | 0 | 0 | 0 | 0 | 0 | 0 | 0 | 0 | 0 | 0 | 0 | 0 | 0 | 0 | 0 | 0 | 0 | 0 | 0 | 0 | 0 | 0 | 0 | 0 | 0 | 0 |
| *Sphenoderia splendida* Playfair, 1917 | 0 | 0 | 0 | 0 | 39 | 22 | 0 | 0 | 0 | 0 | 0 | 0 | 21 | 25 | 0 | 0 | 130 | 42 | 0 | 0 | 0 | 0 | 75 | 28 | 91 | 25 | 0 | 0 | 38 | 23 | 30 | 17 | 17 | 17 | 66 | 38 |
| *Trachelocorythion pulchellum* (Penard, 1890) Bonnet, 1979 | 10 | 21 | 0 | 0 | 33 | 20 | 0 | 0 | 0 | 0 | 16 | 13 | 24 | 31 | 0 | 0 | 29 | 17 | 0 | 0 | 0 | 0 | 0 | 0 | 19 | 7 | 0 | 0 | 6 | 6 | 0 | 0 | 0 | 0 | 0 | 0 |
| *Tracheleuglypha acolla* Bonnet, Thomas, 1955 | 0 | 0 | 0 | 0 | 7 | 7 | 0 | 0 | 0 | 0 | 0 | 0 | 0 | 0 | 0 | 0 | 0 | 0 | 0 | 0 | 0 | 0 | 0 | 0 | 0 | 0 | 0 | 0 | 0 | 0 | 0 | 0 | 0 | 0 | 0 | 0 |
| *Tracheleuglypha dentata* Deflandre, 1928 | 4 | 7 | 0 | 0 | 111 | 72 | 0 | 0 | 0 | 0 | 62 | 52 | 9 | 18 | 0 | 0 | 17 | 7 | 0 | 0 | 0 | 0 | 27 | 27 | 13 | 13 | 0 | 0 | 34 | 34 | 14 | 14 | 14 | 14 | 105 | 72 |
| *Trigonopyxis arcula* Penard, 1912 | 58 | 46 | 0 | 0 | 193 | 93 | 3 | 3 | 3 | 3 | 41 | 16 | 20 | 15 | 0 | 0 | 59 | 16 | 0 | 0 | 0 | 0 | 170 | 71 | 12 | 7 | 0 | 0 | 30 | 11 | 0 | 0 | 0 | 0 | 108 | 53 |
| *Trigonopyxis arcula major* Chardez, 1960 | 13 | 25 | 0 | 0 | 131 | 43 | 0 | 0 | 0 | 0 | 19 | 9 | 36 | 32 | 0 | 0 | 69 | 32 | 0 | 0 | 0 | 0 | 58 | 21 | 26 | 15 | 0 | 0 | 18 | 12 | 0 | 0 | 0 | 0 | 17 | 17 |
| *Trigonopyxis microstoma* Hoogenraad, Groot, 1948 | 0 | 0 | 0 | 0 | 0 | 0 | 0 | 0 | 0 | 0 | 3 | 3 | 0 | 0 | 0 | 0 | 0 | 0 | 0 | 0 | 0 | 0 | 8 | 8 | 37 | 37 | 0 | 0 | 41 | 17 | 0 | 0 | 0 | 0 | 0 | 0 |
| *Trinema chardezi* Decloitre, 1981 | 0 | 0 | 0 | 0 | 0 | 0 | 0 | 0 | 0 | 0 | 0 | 0 | 0 | 0 | 0 | 0 | 8 | 8 | 0 | 0 | 0 | 0 | 0 | 0 | 0 | 0 | 0 | 0 | 13 | 13 | 0 | 0 | 0 | 0 | 14 | 14 |
| *Trinema complanatum* Penard, 1890 | 62 | 44 | 0 | 0 | 123 | 99 | 0 | 0 | 0 | 0 | 0 | 0 | 40 | 30 | 0 | 0 | 128 | 54 | 0 | 0 | 0 | 0 | 0 | 0 | 0 | 0 | 0 | 0 | 0 | 0 | 0 | 0 | 0 | 0 | 0 | 0 |
| *Trinema complanatum* *elongata* Decloitre, 1973 | 0 | 0 | 0 | 0 | 44 | 30 | 0 | 0 | 0 | 0 | 34 | 34 | 25 | 31 | 0 | 0 | 57 | 36 | 0 | 0 | 0 | 0 | 35 | 13 | 32 | 19 | 0 | 0 | 71 | 38 | 0 | 0 | 0 | 0 | 80 | 47 |
| *Trinema complanatum* *inaequalis* Decloitre, 1969 | 0 | 0 | 0 | 0 | 0 | 0 | 0 | 0 | 0 | 0 | 0 | 0 | 0 | 0 | 0 | 0 | 0 | 0 | 0 | 0 | 0 | 0 | 0 | 0 | 0 | 0 | 0 | 0 | 7 | 7 | 0 | 0 | 0 | 0 | 0 | 0 |
| *Trinema enchelys* Leidy, 1878 | 54 | 79 | 0 | 0 | 162 | 53 | 0 | 0 | 0 | 0 | 72 | 33 | 33 | 14 | 0 | 0 | 141 | 29 | 0 | 0 | 0 | 0 | 175 | 87 | 80 | 31 | 0 | 0 | 176 | 56 | 14 | 14 | 32 | 19 | 299 | 116 |
| *Trinema grandis* (Chardez, 1960) Golemansky, 1963 | 21 | 42 | 0 | 0 | 125 | 73 | 0 | 0 | 0 | 0 | 23 | 19 | 41 | 14 | 0 | 0 | 96 | 14 | 13 | 13 | 0 | 0 | 95 | 39 | 37 | 37 | 0 | 0 | 25 | 18 | 0 | 0 | 17 | 17 | 31 | 31 |
| *Trinema lincostoma* Decloitre, 1962 | 0 | 0 | 0 | 0 | 10 | 10 | 0 | 0 | 0 | 0 | 0 | 0 | 0 | 0 | 0 | 0 | 0 | 0 | 0 | 0 | 0 | 0 | 0 | 0 | 0 | 0 | 0 | 0 | 0 | 0 | 0 | 0 | 0 | 0 | 0 | 0 |
| *Trinema lineare* Penard, 1890 | 70 | 27 | 10 | 10 | 174 | 84 | 0 | 0 | 0 | 0 | 8 | 5 | 94 | 71 | 0 | 0 | 264 | 122 | 0 | 0 | 0 | 0 | 144 | 111 | 71 | 44 | 0 | 0 | 115 | 60 | 0 | 0 | 0 | 0 | 94 | 73 |
| *Trinema lineare minuscula* Chardez, 1968 | 25 | 30 | 0 | 0 | 116 | 32 | 0 | 0 | 0 | 0 | 3 | 3 | 37 | 42 | 0 | 0 | 116 | 59 | 0 | 0 | 0 | 0 | 13 | 13 | 43 | 36 | 0 | 0 | 16 | 16 | 0 | 0 | 0 | 0 | 15 | 15 |
| *Trinema lineare* *terricola* Decloitre, 1964 | 4 | 7 | 0 | 0 | 35 | 24 | 0 | 0 | 0 | 0 | 0 | 0 | 4 | 9 | 0 | 0 | 37 | 23 | 0 | 0 | 0 | 0 | 0 | 0 | 0 | 0 | 0 | 0 | 0 | 0 | 0 | 0 | 0 | 0 | 0 | 0 |
| *Trinema lineare* *truncatum* Chardez, 1968 | 0 | 0 | 0 | 0 | 7 | 7 | 0 | 0 | 0 | 0 | 3 | 3 | 0 | 0 | 0 | 0 | 0 | 0 | 0 | 0 | 0 | 0 | 0 | 0 | 12 | 12 | 0 | 0 | 18 | 6 | 0 | 0 | 0 | 0 | 32 | 32 |
| *Trinema penardi* Thomas, Chardez, 1958 | 39 | 44 | 0 | 0 | 124 | 54 | 0 | 0 | 0 | 0 | 3 | 3 | 0 | 0 | 0 | 0 | 38 | 5 | 0 | 0 | 0 | 0 | 0 | 0 | 0 | 0 | 0 | 0 | 7 | 7 | 0 | 0 | 0 | 0 | 15 | 15 |
| Sp. Nov 1 | 0 | 0 | 0 | 0 | 29 | 24 | 3 | 3 | 0 | 0 | 32 | 26 | 0 | 0 | 0 | 0 | 20 | 8 | 0 | 0 | 0 | 0 | 29 | 17 | 0 | 0 | 0 | 0 | 0 | 0 | 0 | 0 | 0 | 0 | 0 | 0 |

SD - Standard Deviation.
