# Supplementary material for: Consequences of exclusion of precipitation on microorganisms and microbial consumers in montane tropical rainforests
Source: Oecologia. 2012 May 22;170(4):1067–76. doi: 10.1007/s00442-012-2360-6 (PMC3496542; doi:10.1007/s00442-012-2360-6)
Supplement: Supplementary file 2 — Supplementary material 2 (DOC 133 kb) [file 442_2012_2360_MOESM2_ESM.doc]

**Consequences of exclusion of precipitation on microorganisms and microbial** **consumers in montane tropical rainforests**

Valentyna Krashevska, Dorothee Sandmann, Mark Maraun and Stefan Scheu

J.F. Blumenbach Institute of Zoology and Anthropology, Georg August University Göttingen, Berliner Straße 28, 37073 Göttingen, Germany

Author for correspondence

Valentyna Krashevska

Mail [vkrashe@gwdg.de](mailto:valentyna.krashevska@biologie.uni-goettingen.de)

Tel: +49 (0) 551 395557

Fax: +49 (0) 551 395448

**Online resource 2.** Variations in the abundance of live cells of testate amoeba taxa with rain exclusion and altitude in tropical montane rainforests (fitted individually; protected ANOVAs) and correlations with biotic and abiotic factors associated with rain exclusion and altitude (Pearson correlation). For authorities of species, see online resource 1.

| Taxa | independent variables | | | | | | environmental factors | | | | | | | | | | | | | |
| --- | --- | --- | --- | --- | --- | --- | --- | --- | --- | --- | --- | --- | --- | --- | --- | --- | --- | --- | --- | --- |
|  | rain  exclusion | | altitude | | rain exclusion  ×  altitude | | basal  respiration | | microbial  biomass | | ergosterol | | water  content | | C-to-N  ratio | | light  intensity | | pH | |
|  | *F* | *p* | *F* | *p* | *F* | *p* | *r* | *p* | *r* | *p* | *r* | *p* | *r* | *p* | *r* | *p* | *r* | *p* | *r* | *p* |
| *Ag. caudata* | 3.3 | 0.09 | 1.03 | 0.38 | 1.03 | 0.38 | 0.34 | 0.1 | 0.45 | 0.03 | 0.36 | 0.09 | 0.35 | 0.1 | 0.05 | 0.81 | 0.37 | 0.08 | -0.17 | 0.43 |
| *Ar. flavum* | 0.18 | 0.67 | 2.45 | 0.11 | 2.08 | 0.15 | 0.19 | 0.38 | -0.11 | 0.59 | 0.32 | 0.12 | 0.28 | 0.18 | 0.34 | 0.1 | 0.37 | 0.05 | 0.07 | 0.73 |
| *As. muscorum* | 3.8 | 0.07 | 5.33 | 0.02 | 1.71 | 0.21 | 0.41 | 0.04 | 0.36 | 0.08 | 0.61 | 0.001 | 0.54 | 0.01 | 0.28 | 0.19 | 0.9 | 0.001 | -0.39 | 0.06 |
| *Ce. plagiostoma* | 1 | 0.33 | 1 | 0.39 | 1 | 0.39 | 0.33 | 0.11 | -0.41 | 0.05 | -0.14 | 0.5 | -0.15 | 0.47 | -0.23 | 0.28 | -0.08 | 0.71 | 0.07 | 0.73 |
| *Cd. oviformis fusca* | 7.25 | 0.01 | 3.64 | 0.05 | 3.64 | 0.05 | 0.44 | 0.03 | 0.42 | 0.04 | 0.53 | 0.01 | 0.55 | 0.01 | 0.13 | 0.55 | 0.81 | 0.001 | -0.27 | 0.2 |
| *Cr. martiali* | 3.95 | 0.05 | 1.55 | 0.24 | 1.55 | 0.24 | 0.37 | 0.08 | 0.45 | 0.03 | 0.4 | 0.05 | 0.47 | 0.02 | 0.15 | 0.5 | 0.63 | 0.001 | -0.24 | 0.26 |
| *Cy. eurystoma* | 5.82 | 0.03 | 0.57 | 0.58 | 0.57 | 0.58 | 0.26 | 0.22 | 0.42 | 0.04 | 0.001 | 0.98 | 0.05 | 0.81 | -0.2 | 0.35 | 0.22 | 0.3 | 0.07 | 0.76 |
| *Cy. eurystoma parvula* | 15.4 | 0.001 | 2.9 | 0.08 | 2.9 | 0.08 | 0.26 | 0.22 | 0.21 | 0.33 | -0.19 | 0.37 | -0.02 | 0.91 | -0.3 | 0.15 | -0.06 | 0.76 | 0.17 | 0.42 |
| *Cy. lithostoma* | 1.27 | 0.28 | 2.39 | 0.12 | 1.27 | 0.31 | 0.18 | 0.4 | 0.04 | 0.84 | -0.22 | 0.29 | -0.3 | 0.15 | -0.37 | 0.05 | -0.11 | 0.6 | 0.22 | 0.31 |
| *Di. lucida* | 1 | 0.33 | 1 | 0.39 | 1 | 0.39 | 0.25 | 0.24 | 0.13 | 0.55 | 0.36 | 0.09 | 0.29 | 0.17 | 0.07 | 0.74 | 0.87 | 0.001 | -0.2 | 0.34 |
| *E. ciliata* | 1 | 0.33 | 1 | 0.39 | 1 | 0.39 | 0.26 | 0.23 | 0.42 | 0.05 | 0.31 | 0.13 | 0.26 | 0.21 | 0.14 | 0.53 | 0.22 | 0.31 | -0.23 | 0.28 |
| *E. laevis* | 21.8 | 0.001 | 3.69 | 0.05 | 3.46 | 0.05 | 0.27 | 0.2 | 0.25 | 0.24 | -0.18 | 0.4 | 0.09 | 0.67 | -0.26 | 0.23 | -0.14 | 0.52 | 0.2 | 0.35 |
| *E. rotunda* | 10.1 | 0.01 | 3.62 | 0.05 | 3.62 | 0.05 | 0.33 | 0.12 | 0.03 | 0.89 | -0.25 | 0.24 | 0.2 | 0.34 | -0.15 | 0.49 | -0.17 | 0.44 | 0.16 | 0.46 |
| *E. simplex* | 6.36 | 0.02 | 6.04 | 0.01 | 7.09 | 0.01 | 0.26 | 0.22 | 0.11 | 0.61 | -0.25 | 0.23 | -0.32 | 0.12 | -0.42 | 0.04 | -0.15 | 0.47 | 0.21 | 0.33 |
| *E. strigosa* | 4.36 | 0.05 | 1.64 | 0.22 | 0.34 | 0.72 | 0.32 | 0.13 | 0.27 | 0.21 | 0.4 | 0.05 | 0.46 | 0.02 | 0.28 | 0.19 | 0.45 | 0.03 | -0.32 | 0.13 |
| *E.* sp2 | 1 | 0.33 | 1 | 0.39 | 1 | 0.39 | 0.25 | 0.24 | 0.13 | 0.55 | 0.36 | 0.09 | 0.29 | 0.17 | 0.07 | 0.74 | 0.87 | 0.001 | -0.2 | 0.34 |
| *He. petricola* | 6.4 | 0.02 | 0.06 | 0.94 | 0.06 | 0.94 | 0.28 | 0.19 | 0.34 | 0.11 | 0.08 | 0.7 | 0.18 | 0.4 | -0.11 | 0.6 | 0.11 | 0.62 | 0.01 | 0.98 |
| *He. rosea* | 4.06 | 0.05 | 4.06 | 0.04 | 4.06 | 0.04 | 0.39 | 0.06 | 0.5 | 0.01 | 0.52 | 0.01 | 0.43 | 0.04 | 0.17 | 0.42 | 0.63 | 0.001 | -0.33 | 0.12 |
| *He. sylvatica* | 1.63 | 0.22 | 2.67 | 0.1 | 4.75 | 0.02 | 0.37 | 0.07 | 0.39 | 0.06 | 0.5 | 0.01 | 0.38 | 0.06 | 0.4 | 0.05 | 0.27 | 0.21 | -0.36 | 0.09 |
| *Hy. subflava* | 6.57 | 0.02 | 3.33 | 0.06 | 0.53 | 0.6 | 0.3 | 0.16 | 0.01 | 0.96 | 0.09 | 0.69 | 0.48 | 0.02 | 0.09 | 0.68 | 0.24 | 0.26 | -0.14 | 0.51 |
| *Hy. subflava* big | 1.89 | 0.19 | 2.55 | 0.11 | 2.22 | 0.14 | 0.17 | 0.44 | -0.02 | 0.91 | 0.08 | 0.7 | 0.41 | 0.05 | 0.13 | 0.54 | -0.03 | 0.88 | -0.09 | 0.66 |
| *N. collaris* | 3.02 | 0.1 | 1.59 | 0.23 | 1.59 | 0.23 | 0.34 | 0.1 | 0.35 | 0.1 | 0.44 | 0.03 | 0.43 | 0.04 | 0.32 | 0.13 | 0.31 | 0.15 | -0.27 | 0.19 |
| *N. militaris* | 4.42 | 0.05 | 0.41 | 0.67 | 0.41 | 0.67 | 0.14 | 0.53 | 0.02 | 0.91 | -0.04 | 0.84 | 0.09 | 0.68 | -0.12 | 0.56 | 0.03 | 0.88 | 0.05 | 0.82 |
| *N. tincta* | 8.61 | 0.01 | 3.66 | 0.05 | 3.66 | 0.05 | 0.48 | 0.02 | 0.34 | 0.11 | 0.51 | 0.01 | 0.57 | 0.001 | 0.31 | 0.15 | 0.66 | 0.001 | -0.33 | 0.11 |
| *Pa. tubulata* | 3.56 | 0.08 | 1.01 | 0.38 | 1.01 | 0.38 | 0.38 | 0.06 | 0.09 | 0.69 | 0.3 | 0.16 | 0.43 | 0.04 | 0.26 | 0.22 | 0.37 | 0.08 | -0.22 | 0.3 |
| *Pg. declivis* | 4.89 | 0.04 | 1.83 | 0.19 | 1.83 | 0.19 | 0.36 | 0.08 | 0.32 | 0.13 | -0.22 | 0.3 | -0.12 | 0.57 | -0.34 | 0.1 | -0.15 | 0.48 | 0.2 | 0.35 |
| *Ph. paradoxa* | 2.25 | 0.15 | 2.07 | 0.16 | 0.28 | 0.76 | 0.32 | 0.12 | 0.42 | 0.04 | 0.47 | 0.02 | 0.25 | 0.24 | 0.02 | 0.94 | 0.45 | 0.03 | -0.3 | 0.15 |
| *Pl. spinosa* | 1.79 | 0.2 | 1.79 | 0.2 | 1.79 | 0.2 | 0.31 | 0.13 | 0.21 | 0.32 | 0.21 | 0.32 | 0.33 | 0.12 | 0.22 | 0.31 | 0.45 | 0.03 | -0.29 | 0.17 |
| *Ps. gracilis* | 5.15 | 0.04 | 1.41 | 0.27 | 1.41 | 0.27 | 0.41 | 0.05 | 0.17 | 0.42 | 0.36 | 0.08 | 0.51 | 0.01 | 0.26 | 0.23 | 0.59 | 0.001 | -0.21 | 0.32 |
| *S. fissirostris* | 3.57 | 0.08 | 1.02 | 0.38 | 1.02 | 0.38 | 0.38 | 0.06 | 0.16 | 0.47 | 0.29 | 0.17 | 0.45 | 0.03 | 0.11 | 0.6 | 0.58 | 0.001 | -0.16 | 0.46 |
| *S. splendida* | 6.2 | 0.02 | 11.5 | 0.001 | 2.7 | 0.09 | 0.49 | 0.02 | 0.53 | 0.01 | 0.72 | 0.001 | 0.63 | 0.001 | 0.44 | 0.03 | 0.52 | 0.01 | -0.53 | 0.01 |
| *Th. pulchellum* | 7.46 | 0.01 | 0.36 | 0.7 | 0.36 | 0.7 | 0.32 | 0.12 | 0.21 | 0.32 | 0.18 | 0.4 | 0.37 | 0.08 | 0.05 | 0.81 | 0.08 | 0.71 | -0.1 | 0.65 |
| *Tg. arcula* | 12.1 | 0.001 | 3.18 | 0.07 | 2.51 | 0.11 | 0.54 | 0.01 | 0.34 | 0.11 | -0.18 | 0.4 | -0.1 | 0.64 | -0.4 | 0.05 | 0.03 | 0.87 | 0.18 | 0.4 |
| *Tg. arcula major* | 8.61 | 0.01 | 0.65 | 0.53 | 0.65 | 0.53 | 0.48 | 0.02 | 0.21 | 0.33 | 0.14 | 0.5 | 0.39 | 0.06 | 0.12 | 0.59 | -0.01 | 0.96 | -0.1 | 0.66 |
| *Tg. microstoma* | 1 | 0.33 | 1 | 0.39 | 1 | 0.39 | 0.25 | 0.24 | 0.13 | 0.55 | 0.36 | 0.09 | 0.29 | 0.17 | 0.07 | 0.74 | 0.87 | 0.001 | -0.2 | 0.34 |
| *Tr. complanatum* | 14.9 | 0.001 | 4.24 | 0.03 | 4.24 | 0.03 | 0.39 | 0.06 | 0.21 | 0.34 | -0.26 | 0.22 | -0.1 | 0.64 | -0.36 | 0.09 | -0.18 | 0.4 | 0.21 | 0.33 |
| *Tr. compl. elongata* | 5.4 | 0.03 | 1.4 | 0.27 | 1.4 | 0.27 | 0.36 | 0.08 | 0.39 | 0.06 | 0.33 | 0.12 | 0.49 | 0.01 | 0.24 | 0.27 | 0.1 | 0.65 | -0.22 | 0.31 |
| *Tr. enchelys* | 8.5 | 0.01 | 1.05 | 0.37 | 0.29 | 0.75 | 0.41 | 0.04 | 0.47 | 0.02 | 0.4 | 0.06 | 0.32 | 0.13 | 0.15 | 0.48 | 0.24 | 0.26 | -0.26 | 0.22 |
| *Tr. grandis* | 3.61 | 0.07 | 0.42 | 0.67 | 0.1 | 0.91 | 0.29 | 0.17 | 0.33 | 0.12 | 0.15 | 0.49 | 0.3 | 0.16 | 0.1 | 0.65 | 0.06 | 0.78 | -0.1 | 0.64 |
| *Tr. lineare* | 16.1 | 0.001 | 0.16 | 0.85 | 0.16 | 0.85 | 0.46 | 0.02 | 0.32 | 0.13 | 0.12 | 0.57 | 0.35 | 0.09 | 0.03 | 0.88 | -0.07 | 0.75 | -0.06 | 0.78 |
| *Tr. lineare minuscula* | 5.78 | 0.03 | 0.14 | 0.87 | 0.14 | 0.87 | 0.3 | 0.16 | 0.17 | 0.42 | 0.23 | 0.28 | 0.35 | 0.09 | -0.04 | 0.84 | 0.66 | 0.001 | -0.08 | 0.7 |
| *Tr. penardi* | 3.13 | 0.09 | 3.13 | 0.07 | 3.13 | 0.07 | 0.22 | 0.3 | 0.14 | 0.51 | -0.17 | 0.42 | -0.3 | 0.16 | -0.38 | 0.05 | -0.11 | 0.61 | 0.16 | 0.45 |
| Sp. Nov 1 | 1 | 0.33 | 1 | 0.39 | 1 | 0.39 | -0.31 | 0.1 | -0.28 | 0.18 | -0.28 | 0.19 | -0.4 | 0.05 | -0.18 | 0.39 | -0.09 | 0.68 | 0.31 | 0.14 |
